# Glomerulocapillary miRNA response to HLA-class I antibody *in vitro* and *in vivo*

**DOI:** 10.1038/s41598-017-14674-5

**Published:** 2017-11-06

**Authors:** Falko M. Heinemann, Peter T. Jindra, Clemens L. Bockmeyer, Philip Zeuschner, Juliane Wittig, Heike Höflich, Marc Eßer, Mahmoud Abbas, Georg Dieplinger, Katharina Stolle, Udo Vester, Peter F. Hoyer, Stephan Immenschuh, Andreas Heinold, Peter A. Horn, Wentian Li, Ute Eisenberger, Jan U. Becker

**Affiliations:** 1 Institute for Transfusion Medicine, University Hospital Essen, University Duisburg-Essen, Essen, Germany; 2 Immune Evaluation Laboratory, Department of Surgery, Baylor College of Medicine, Houston, TX USA; 3 Institute of Pathology, Department of Nephropathology, University Hospital Erlangen-Nürnberg, Erlangen, Germany; 4 Institute of Pathology, University Hospital of Cologne, Cologne, Germany; 5 Institute of Pathology, Hannover Medical School, Hannover, Germany; 6 PATHOCOM, Rheine, Germany; 7 Department of General, Visceral and Cancer Surgery, Transplant Center Cologne, University of Cologne, Cologne, Germany; 8 Children’s Hospital, Pediatrics II, University of Duisburg-Essen, Essen, Germany; 9 Institute of Transfusion Medicine, Hannover Medical School, Hannover, Germany; 10 Robert S Boas Center for Genomics and Human Genetics, Feinstein Institute for Medical Research, Northwell Health, Manhasset, NY USA; 11 Clinic for Nephrology, University Hospital Essen, University Duisburg-Essen, Essen, Germany

## Abstract

Changes in miRNA expression of glomerular capillaries during antibody-mediated rejection (ABMR) are poorly understood and could contribute to the deleterious inflammation and fibrosis of ABMR via suppression of target genes. A better understanding could lead to novel diagnostic tools and reveal novel therapeutic targets. We explored deregulated miRNAs in an glomeruloendothelial *in vitro* model of ABMR due to class I human leukocyte antigen (HLA) with and without complement activation. We studied a set of 16 promising candidate miRNAs in microdissected glomeruli a confirmation set of 20 human transplant biopsies (DSA+) compared to 10 matched controls without evidence for ABMR. Twelve out of these 16 glomerulocapillary miRNAs could successfully be confirmed as dysregulated *in vivo* with 10 upregulated (let-7c-5p, miR-28-3p, miR-30d-5p, miR-99b-5p, miR-125a-5p, miR-195-5p, miR-374b-3p, miR-484, miR-501-3p, miR-520e) and 2 downregulated (miR29b-3p, miR-885-5p) in DSA+ vs. controls. A random forest analysis based on glomerular miRNAs identified 18/20 DSA+ and 8/10 controls correctly. This glomerulocapillary miRNA signature associated with HLA class I-DSA could improve our understanding of ABMR and be useful for diagnostic or therapeutic purposes.

## Introduction

The main cause for chronic kidney transplant loss besides recurrence of the primary disease is antibody-mediated rejection (ABMR)^1,2^. The diagnosis of acute ABMR is challenging, with current Banff guidelines resting on four cornerstones: 1) donor-specific antibody (DSA), particularly against human leukocyte antigen (HLA), 2) the vascular lesions endarteritis, thrombotic microangiopathy, glomerular and peritubular capillaritis, 3) C4d positivity of endothelial cells and 4) mRNA expression profiling^3^. In clinical practice, fulfilment of the criteria is often not possible due to the varying specificity and sensitivity of the respective test. Moreover, the pathogenesis of endothelial damage or activation is far from understood, despite recent advances in the investigation of endothelial changes upon anti-HLA-class I alloantibody binding with and without complement activation. miRNAs are regulators of mRNA levels and mRNA translation^4^. As small RNA fragments of about 20 nucleotides in length they suppress whole pathways^5^ and are involved in pathologic processes like cell death^6^, inflammation^7^ and fibrosis^8^.

As a first step towards an improved understanding of miRNA changes in endothelial cells secondary to HLA-class I DSA and to identify diagnostic glomerulocapillary miRNA expression signatures, we investigated an *in vitro* model of anti-HLA-class I ABMR with and without complement activation which was based on a standard diagnostic test recommended by the American Society For Histocompatibility and Immunogenetics^9^. In a retrospective pilot study, candidate miRNAs were confirmed for their *in vivo* relevance in microdissected glomeruli from human transplants with anti-HLA-class I DSA and compared to matched controls. The target pathways of deregulated glomerular miRNAs were explored *in silico*.

## Results

### Differential expression of miRNAs *in vitro*

**Figure 1:**
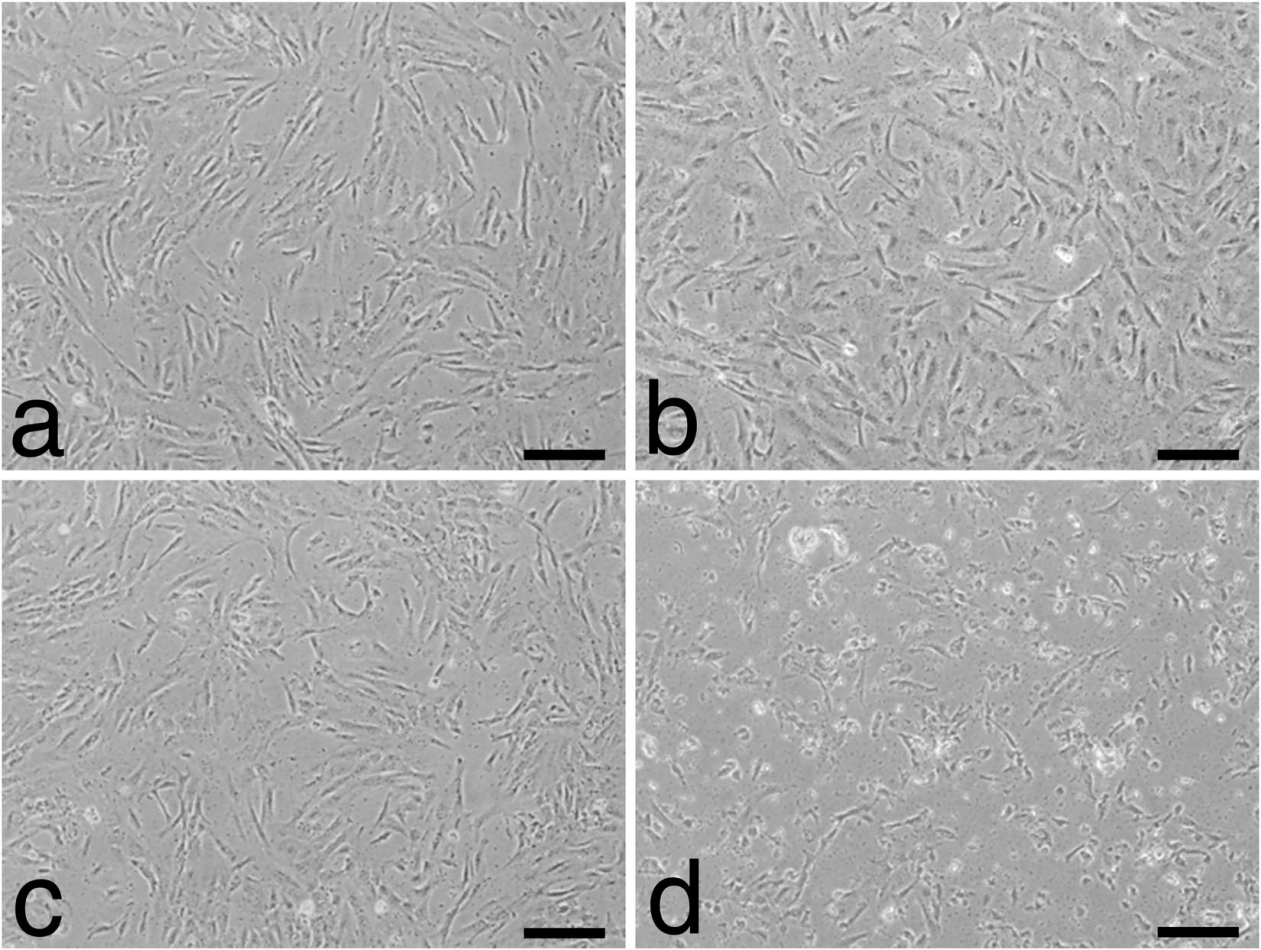
Representative micrographs of human glomerular endothelial cells in the *in vitro* model of anti-HLA class I-mediated ABMR. After incubation with (**a**) irrelevant anti-A1 and without complement, (**b**) irrelevant anti-A1followed by complement, (**c**) anti-A2 without complement and (**d**) anti-A2 followed by complement. Only the latter showed marked cytopathic changes with cytoplasmic retraction and shrinking.

**Figure 2:**
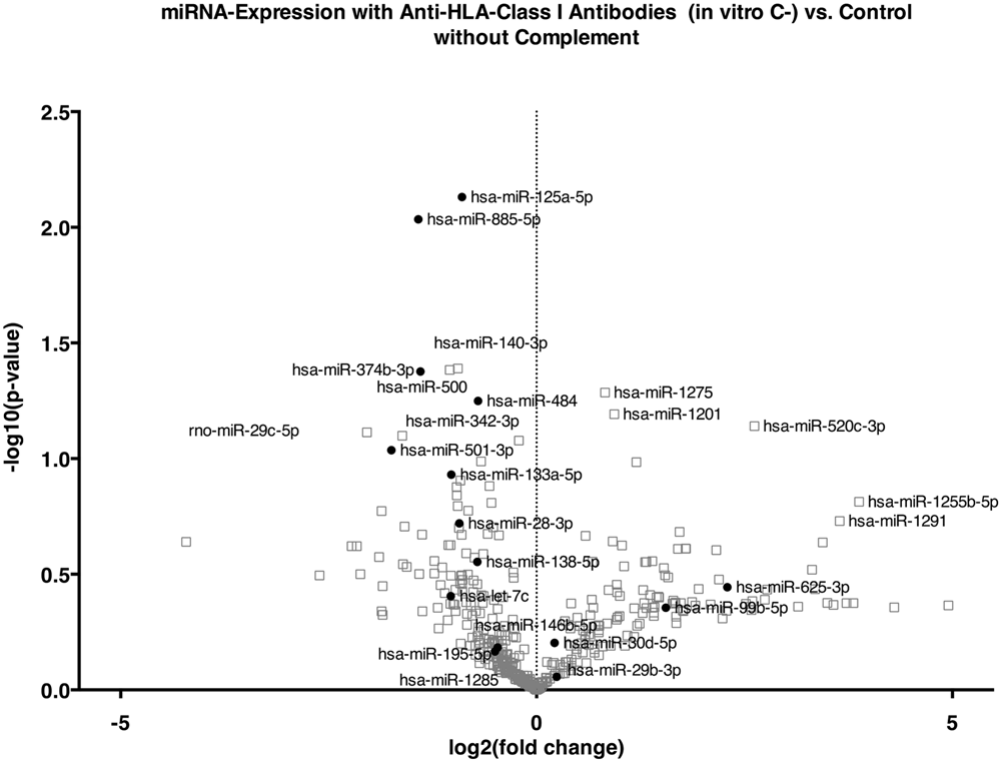
Volcano plot of differentially regulated miRNAs *in vitro* upon stimulation of endothelial cells with anti-HLA-class I antibodies without complement. The x-axis shows the log2 of the fold change between *in vitro* C− and the control, the y-axis shows the −log10 of the p-value of a two-sided t-test. Each experiment was performed in triplicates. miRNAs that were included in the validation on microdissected glomeruli from transplant biopsies based on differential expression and validated target pathways are shown in solid dots, those excluded in gray squares.

**Figure 3:**
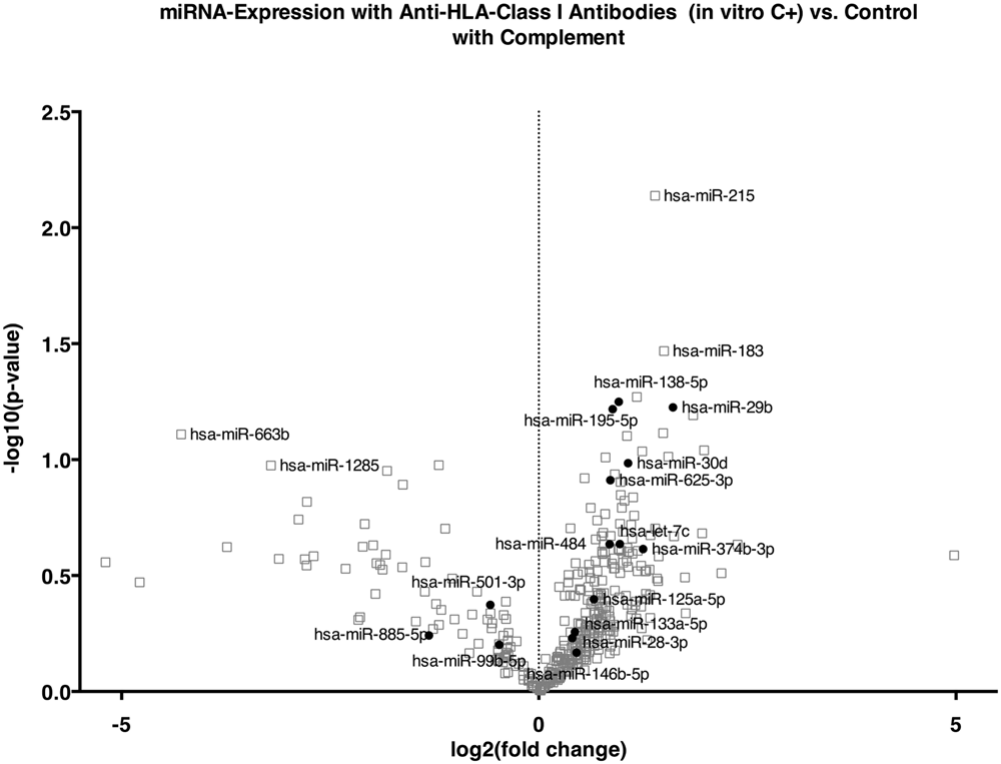
Volcano plot of differentially regulated miRNAs *in vitro* upon stimulation of endothelial cells with anti-HLA-class I antibodies follow by incubation with rabbit complement. The x-axis shows the log2 of the fold change between *in vitro* C+ and the control, the y-axis shows the −log10 of the p-value of a two-sided t-test. Each experiment was performed in triplicates. miRNAs that were included in the validation on microdissected glomeruli from transplant biopsies based on differential expression and validated target pathways are shown in solid dots, those excluded in gray squares.

The cytoplasm showed a spindle to cobblestone shape. Sequential incubation with specific anti-A2 without complement as a model for anti-HLA-class I ABMR without complement activation (*in vitro* C−), incubation with the respective controls irrelevant anti-A1 without complement (*in vitro* control without complement) and with complement (*in vitro* control with complement) did not show significant alterations in cell morphology. Only specific anti-A2 with complement as a model for anti-HLA-class I ABMR with complement activation (*in vitro* C+) caused severe cytoplasmic retraction and shrinking (Fig. 1). All expression data from the *in vitro* model can be downloaded as a supplementary file (TBA). Volcano plots show the differential miRNA expression in the *in vitro* models of complement-independent and HLA-class I DSA complement-binding (Figs 2 and 3). Based on inspection of the volcano plots and an *in silico* analysis of regulated pathways with DIANA miRPath v.2.0^10^ we chose a set of 16 miRNAs for confirmation in microdissected glomeruli from patients with only HLA-class I DSAs: miR-let-7c, miR-28-3p, miR-29b, miR-30d, miR-99b, miR-125a-5p, miR-133a, miR-138, miR-146b, miR-195, miR-374b-3p, miR-484, miR-501-3p, miR-520e, miR-625-3p, miR-885-5p (Table 1).

**Table 1:** Candidate set of 16 miRNAs for validation in the human biopsies.

miR	miRNA-Cluster	*in vitro*		Glomeruli in Biopsy		Selected Validated Targets	miRPath KEGG Target Pathway
		Higher	Lower	Higher	Lower		
**let-7c-5p**	miR-99a	C+	C−	DSA+		TGFBR1, HMGA2, MPL	Apoptosis, PI3K-Akt-Signalling, NF-kappa B Signalling, Cell Cycle, ErbB Signalling, TGF-beta Signalling
**miR-28-3p**	None	C+	C−	DSA+			None found.
**miR-29b-3p**	None	C−			DSA+	TGFB1, TGFB2, TGFB3, HDAC4, COL4A1, COL4A2, COL1A1, SP1, DNMT3A, MCL1, DNMT1, VEGFA, MMP15, GRN, FGA, FGB, FGG, COL3A1, MMP2, ADAM12, HMGA2, CDC42, TBX21, IFNG, SERPINH1, TET2, LAMC2, PDGFA, PDGFB, PDGFC, PDGFRA, MMP9, LOXL4, ITGB1,	ECM-receptor interaction, PI3K-Akt Signalling, Complement and Coagulation Cascades, TGF-beta Signalling, mTOR Signalling, p53 Signalling
**miR-30d-5p**	miR-30b	C+, C−		DSA+		SMAD1, CASP3, TP53, SNAI1, EZH2, RUNX2, SOCS1, NOTCH1, KPNB1, ATG2B, ATG5, ATG12, BCN1	p53 Signalling
**miR-99b-5p**	let-7e miR-125a-5p	C−	C+	DSA+		MTOR, NOX4	Leukocyte Transendothelial Migration, MAPK Signalling
**miR-125a-5p**	let-7e miR-99b-5p	C+	C	DSA+		CDKN1A, LIN28A, CD34, TP53, VEGFA, ERBB2, ERBB3, ELAVL1, TRAF6, SIRT7	ErbB Signalling, HIF-1 Signalling, Focal Adhesion, Insulin Signalling
**miR-133a-5p**	None	C+	C−	n.s.			Targets suchen
**miR-138-5p**	None	C+	C−	n.s.		ROCK2, RHOC, H2AFX, TERT, IGF1R, SIRT1, FOSL1, HIF1A, CASP3, EZH2, ZEB2, VIM, S100A1, SENP1, GPR124,	PPAR Signalling, Cell Adhesion Molecules, Complement and Coagulation Cascades, Leukocyte Transendothelial Migration, p53 Signalling
**miR-146b-5p**	None	C+	C−	n.s.		NFKB1, CDKN1A, TRAF6, TLR4, PDGFRA,	NF-kappa B Signalling, Toll-like Receptor Signalling, Apoptosis, HIF-1 Signalling
**miR-195-5p**	miR-497	C+	C−	DSA+		E2F3, VEGFA, CDC42, BIRC5, ATG14,	PI3K-Akt Signalling, Cell Cycle, p53, Focal Adhesion, Apoptosis, HIF-1 Signalling, NF-kappa B Signalling
**miR-374b-3p**	miR-374c miR-421	C+	C−	DSA+		none given	ErbB Signalling
**miR-484**	None	C+	C−	DSA+		VEGFR2	Cytokine-Cytokine Receptor Interaction
**miR-501-3p**	miR-188 miR-362 miR-500a miR-500b miR-502 miR-532 miR-660		C+, C−	DSA+		none given	MAPK Signalling, PI3K-Akt Signalling,
**miR-520e**	miR-512-1 miR-512-2 miR-1323 miR-498 miR-515-1 miR-515-2 miR-519e miR-520f	C+*		DSA+		CD46	Complement and Coagulation Cascades
**miR-625-3p**	None	C+, C−		n.s.		none given	MAPK Signalling
**miR-625-3p**	None		C+, C−		DSA+	CASP3	Cell Cycle, p53 Signalling

The candidates were chosen after visual inspection of the volcano plots in Figs 1 and 2 under consideration of the validated targets and pathways according to miRPath. Although miR-520e was only detected in one sample of *in vitro* C+, it was included among the validation set, because of the validated target CD46, which is a complement regulator.

The columns show whether the respective miRNA was higher or lower than controls in the *in vitro* experiments or in validation in glomeruli of transplant biopsies with HLA-class I DSA (DSA+) or controls. Selected validated targets are given with their gene symbols and the last columns lists all target pathways according to MirPath. The references to the validated targets can be found in the Discussion, if not mentioned there, they were derived from miRPath. Abbreviations: C+ (with complement), C− (without complement).

### Clinical and histological findings of the confirmation set of human renal transplant biopsies

Clinical findings of patients are given in Table 2. Except for a higher proportion of living donors in the control cohort (6/10 vs. 3/20; p = 0.030) which should not be a confounder at this time after transplantation, we could not find a significant difference between the cohorts. Further splitting of the DSA+ cohort into subcohorts was avoided due to insufficient sample sizes for pairwise comparisons. Each two biopsies of the DSA+ and the control cohorts were derived from AB0-incompatible transplants, the rest from AB0-compatible transplants. All DSA+ patients had *de novo* DSA.

**Table 2:** Clinical data of the 20 patients with HLA-class I DSA (DSA+) and the 10 controls.

	HLA Class I DSA-positive Patients (DSA+) n = 20	Controls n = 10	P
**Patient Sex**	9 Female, 11 Male	4 Female, 6 Male	1
**Primary Disease**	ADPKD 3	ADPKD 2	0.554
	Atherosclerotic Nephropathy 1	Atherosclerotic Nephropathy 0	
	Alport Syndrome 1	Alport Syndrome 0	
	Crush Injury 0	Crush Injury 1	
	Diabetic Nephropathy 1	Diabetic Nephropathy 1	
	Goodpasture Syndrome 1	Goodpasture Syndrome 0	
	GPA 1	GPA 0	
	GN NOS 2	GN NOS 1	
	HUS 3	HUS 0	
	Hypertensive Nephropathy 1	Hypertensive Nephropathy 1	
	IgA-GN 1	IgA-GN 0	
	Primary FSGS 1	Primary FSGS 1	
	Pyelonephritis 0	Pyelonephritis 1	
	Unknown 4	Unknown 2	
**Number of Previous Transplants**	No Transplant 17	No Transplant 10	0.246
	One Transplant 2		
	Three Transplants 1		
**Other Transplants**	0	1 (Pancreas)	0.313
**Donor Sex**	10 female, 10 male	1 female, 8 male*	0.096
**Donor Age in Years**	45 (29; 58)	49 (21; 66)*	0.931
**Donor Type**	3 living, 17 deceased	6 living, 4 deceased	0.03
**AB0 compatible/incompatible**	18 compatible, 2 incompatible	8 compatible, 2 incompatible	0.584
**Patient Age at Biopsy in Years**	48 (38; 57)	49 (40; 58)	0.877
**Weeks since Transplantation**	31 (11; 77)	51 (21; 182)	0.454
eGFR at Biopsy in ml/(min*1.73m ^2^ **)**	26 (19; 34)	27 (20; 44)	0.691

The only significant difference between the two cohorts was found in the proportion of living donor transplants, which were more frequent in the controls. All numerical data are given as the median and the interquartile range (IQR).

Abbreviations: autosomal dominant polycystic kidney disease (ADPKD), estimated glomerular filtration rate (eGFR), focal and segmental glomerulosclerosis (FSGS), glomerluonephritis (GN), granulomatosis with polyangiitis (GPA), hemolytic-uremic syndrome (HUS), not otherwise specified (NOS).

^*^n = 9 for the controls, because data were not available for one patient transplanted in 1992.

**Figure 4:**
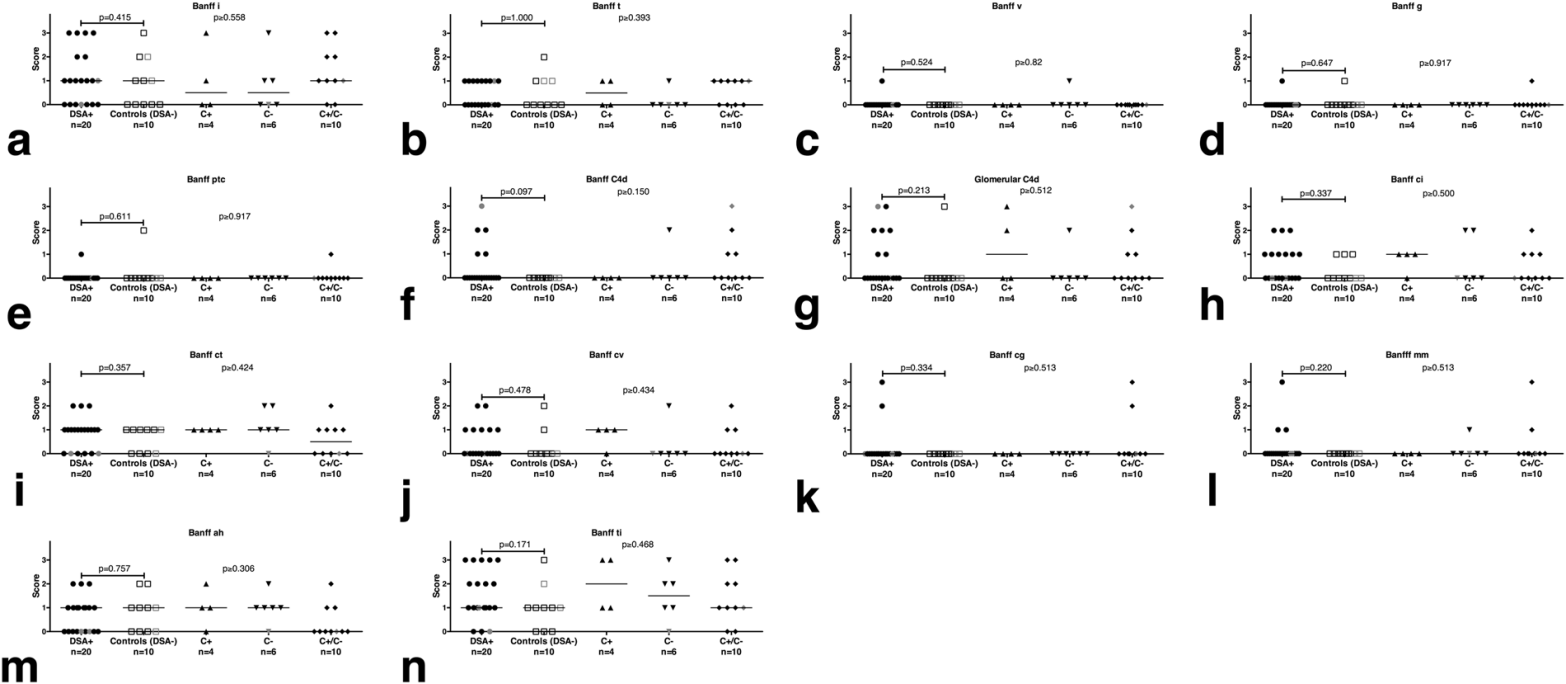
Banff components and glomerular C4d scores of all subgroups of patients with HLA-class I DSA (DSA+). These include only complement-binding (C+), only non-complement-binding (C−), both complement-binding and non-complement-binding (C+/C−). All biopsies from an AB0-incompatible transplant are shown in grey, the others in black. The p-value above the bracket between DSA+ and controls relates to a Wilcoxon test between these two cohorts; the p-value on the right relates to a non-parametric pairwise comparison (Steel-Dwass) between controls, C+, C− and C+/C−. For none of the individual components could we find a significant difference in the comparison between DSA+ and controls and in the pairwise comparisons between the DSA+ subgroups and the controls.

The relevant Banff components and the score for glomerular C4d for all control and DSA+ biopsies are shown in Fig. 4. We could not find a significant difference between DSA+ patients and controls for these components.

### Confirmation of differentially expressed glomerular miRNAs in human transplant biopsies

**Figure 5:**
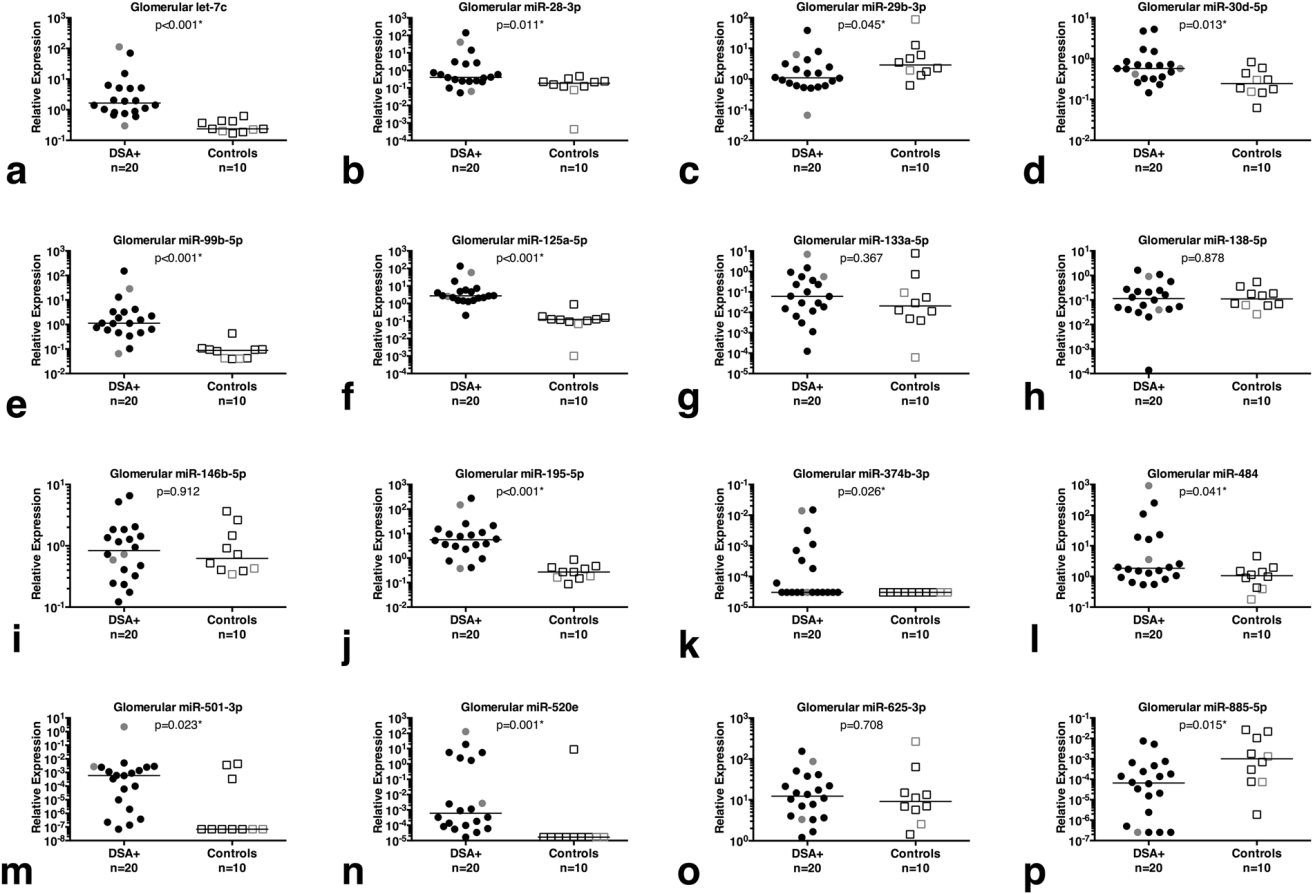
Differential expression of glomerular miRNAs in human transplant biopsies with HLA class I-DSA (DSA+) vs. controls. All AB0-incompatible transplants are shown in grey, the others in black. The bar represents the median of each cohort. Glomerular miR-let-7c-5p (**a**), miR-28-3p (**b**), miR-30d-5p (**d**), miR-99b-5p (**e**), miR-125a-5p (**f**) and miR-195-5p (**j**), miR-374b-3p (**k**), miR-484 (**l**), miR-501-3p (**m**), miR-520e (**n**) and miR-885-5p (**p**) were higher in DSA+ than to controls. Glomerular miR-29b-3p (**c**) and miR-885-5p (**p**) were lower in DSA+ than in controls. We could not find any significant difference for the other four glomerular miRNAs examined.

We decided to retain the each two biopsies from AB0-incompatible transplants (shown as grey symbols in Figs 3, 4 and 5) in the analysis, because we could not find any obvious effect of the AB0 incompatibility on the glomerular expression of the 16 miRNAs examined.

Comparing all DSA+ biopsies with the controls, 12 out of the 16 candidate glomerulocapillary miRNAs (75%) were found to be differentially regulated (Fig. 5). All expression values are given as the median followed by the interquartile range (IQR). miR-let-7c was upregulated in DSA+ (median 1.67; IQR 0.83; 5.13 vs. median 0.24; IQR 0.20; 0.43; p < 0.001), so were miR-28-3p (0.40; 0.24; 2.60 vs 0.19; 0.11; 0.27; p = 0.011), miR-30d-5p (0.57; 0.33; 0.81 vs. 0.24; 0.15; 0.48; p = 0.013), miR-99b-5p (1.27; 0.50; 3.29 vs. 0.09; 0.04; 0.10; p < 0.001), miR-125a-5p (2.72; 1.46; 5.14 vs. 0.12; 0.09; 0.16; p < 0.001), miR-195-5p (5.59; 2.48; 14.14 vs. 0.27; 0.16; 0.43; p < 0.001), miR-374b-3p (3.04e-5; 3.04e-5; 6.12e-4 vs. 3.04e-5; 3.04e-5; 3.04e-5; p = 0.026), miR-484 (1.85; 0.96; 18.40 vs. 1.06; 0.42; 1.61; p = 0.046), miR-501-3p (5.96e-4; 3.92e-6; 2.43e-3 vs. 6.89e-9; 6.89e-8; 1.14e-3; p = 0.023), miR-520e (6.1e-4; 8.66e-5; 2.27 vs. 1.68e-5; 1.68e-5; 1.58e-5; p = 0.001). Downregulated in DSA+ were glomerular miR-29b-3p (1.10; 0.60; 2.96 vs. 2.87; 1.164; 7.76; p = 0.045) and miR-885-5p (6.56e-5; 9.83e-7; 4.04e-4 vs. 1.01e-3; 7.54e-5; 1.33e-2; p = 0.015). No significant difference was found for glomerular miR-133a-3p, miR-138-5p, miR-146b-5p or miR-625-3p.

### Correlation with glomerular Banff components

Since only the glomerular C4d score was positive (≥1) in at least 4 biopsies, rendering acceptable group sizes, we examined only the correlation of this parameter with glomerular miRNA expression. Glomerular miR-30d-5p (0.70; 0.49; 1.33 vs. 0.32; 0.19; 0.61; p = 0.018) and miR-885-5p (8.88e-6; 3.16e-7; 0.21e-4 vs. 2.68e-4; 7.42e-7; 1.43e-3; p = 0.041) were both higher in positive than in negative biopsies.

### Random forest analysis of glomerular miRNAs for the diagnosis of anti-HLA-class I DSA

In this pilot study we tried to show the potential of a combined assessment of our candidate glomerular miRNAs as markers of HLA-class I DSA. To this end we performed a random forest analysis for the discrimination of DSA+ (n = 20) vs. controls (n = 10). Of the 20 DSA+ biopsies, 18 were classified correctly using the 16 miRNA expression levels (OOB classification error rate 2/20 = 10%). Of the 10 control samples, 8 were classified correctly (OOB classification error rate 2/10 = 20%). The number of trees used in the analysis was the default value of 500; use of other values instead of the default returned similar values. The top three miRNAs which contributed the most to the classification performance were miR-125a-5p, let-7c-5p, and miR-99b-5p.

## Discussion

This report is the first description of glomerulocapillary miRNA expression in transplants with DSA. With our *in vitro* model of ABMR with HLA-class I-DSA with and without complement activation which was based on a standard immunogenetical diagnostic test, we identified miRNAs that could explain the glomerulocapillary inflammation and remodelling of ABMR and that appear promising for diagnostic use.

Out of the 16 miRNAs that were subjected to confirmation on microdissected glomeruli from DSA+ biopsies, 12 were found to be differentially regulated compared to controls without DSA in our cohort. Only miR-501-3p showed a discrepancy between the *in vitro* and the *in vivo* findings. *In vitro*, this particular miRNA was lower both in C− and in C+, whereas *in vivo* it was higher in microdissected glomeruli of DSA+ compared to the controls. The reason for this discrepancy is unclear. One could speculate that this discrepancy might be due to the different time course. *In vitro*, DSA and complement were effective for less than two hours only. In the human transplants, the DSA would have been effective for a much longer time and a steady state regarding miRNA expression may have been reached.

Our compartment-specific approach adds to the previously discovered miRNAs in whole biopsy tissue with AMBR. With miR-146b-5p only one of the six miRNAs previously described as upregulated in ABMR was among our candidate miRNAs. However, the difference in glomerulocapillary expression between DSA+ and controls did not reach statistical significance for this particular miRNA in our study. There are at least two explanations for this discrepancy: firstly, in contrast to our DSA+ cohort, their ABMR cohort was compiled of C4d-positive cases with histological signs of ABMR, DSA data were not mentioned; secondly, our compartment-specific study examined glomeruli, whereas they analysed whole biopsy cores. With our cohort sizes larger than theirs, a type II error seems less likely but cannot be excluded.

We could find a range of interesting targets for the differentially regulated glomerulocapillary miRNAs, some of which point to novel pathways in ABMR.

The upregulation of let-7c-5p, one of the top three performers in the random forest analysis, in DSA+ biopsies should have a negative effect. It has been described to initiate apoptosis of endothelial cells via targeting of Bcl-xl^11^. Bcl-xl has long been considered an endothelial protective factor in transplantation^12^, particularly in ABMR^13,14^. Interestingly, *in vitro* experiments similar to ours with a mouse monoclonal antibody against HLA-class I without complement have shown upregulation of BCL2L1 independently of PIK3/AKT1 signalling; this finding was confirmed in human heart transplants^15^. The downregulation of let-7c-5p in our *in vitro* experiments without complement could contribute to the induction of BCL2L1 in this setting. Complement reversed the effect and upregulated let-7c-5p. Similarly, the upregulation of let-7c-5p in transplant glomeruli should lead to detrimental downregulation of BCL2L1. Moreover, suppression of HMGA2 by let-7c-5p should be detrimental, because HMGA2 suppression has been shown to promote senescence in endothelial progenitor cells^16^.

miR-28-3p was one of three miRNAs, which were lower in DSA+ than in controls. miRPath did not yield validated targets or pathways. An *in vitro* study in cardiomyocytes describes suppression of the PDPK1/AKT1/MTOR signalling pathway as a function of miR-28-3p. Less suppression of this pathway would result in less activation of allogeneic regulatory T-cells by endothelial cells^16^ and less suppression of proinflammatory VCAM1 in endothelial cells via mTORC2^17^. Thus, less miR-28-3p in DSA+ could have proinflammatory effects.

Similarly, the decreased expression of miR-29b-3p should have a detrimental, procoagulant, proinflammatory and profibrotic effect. miR-29b-3p has been shown to target a host of collagen remodelling factors^18,19^ and profibrotic cytokines^20^ as well as extracellular matrix components relevant in kidney transplant fibrosis, transplant vasculopathy and glomerulopathy, among them MMP2^21,22^, TGFB1^23^, collagen type I, type III and type IV^24^ and LAMC2^25^.

miR-99b-5p and miR-125a-5p both belong to the same cluster and are known to be expressed in endothelial cells^26^. Both were found upregulated in DSA+ glomeruli and were among the top three performing miRNAs in the RF analysis. Both miRNAs are known to activate the proinflammatory^27^ NFkappa-B pathway^28^. Moreover, miR-125a-5p inhibits LIN28A; LIN28A in turn inhibits hsa-let-7c-5p biogenesis^29–31^, which could indirectly contribute to the rise of let-7c-5p we have observed. LIN28A has a role in tissue repair^32^, which would be diminished upon upregulation of miR-125a-5p. Lastly suppression of angiogenesis promoting ERBB2 and ERBB3^33^ could result in a blunted angiogenic response.

Upregulation of miR-195-5p seems to have equivocal effects. It suppresses proangiogenic target E2F3^34,35^. Another target, endothelial CDC42, inhibits various mechanisms of blood vessel formation^36–39^. However, it also inhibits inflammatory activation in senescent endothelial cells. Another target is endothelial apoptosis-inhibitor BIRC5^40^. BIRC5, also involved in angiogenesis^41^, protects microvascular endothelial cells from alloimmune attack^42^.

Another miRNA upregulated in DSA+ was miR-374b-3p; its target NMMLCK contributes to endothelial barrier dysfunction in inflammation^43^. miR-374b-3p also targets RECK^44^, which is important in angiogenesis. Suppression of RECK results in defective angiogenesis and extracellular matrix composition through the lack of inhibition of MMP2 and MMP9^45^.

miR-484 was also upregulated in DSA+, which should have detrimental effects. Targets include VEGFR2^46^, which is expressed in glomerular endothelial cells; activation of VEGFR2 decreased glomerular endothelial barrier function *in vitro*^47^. VEGFR2 activation also results in protective eNOS activation in glomerular endothelial cells^48^.

miR-520e, which was only included in the validation set because of the interesting target complement regulator CD46^49^ was upregulated in glomeruli of DSA+. CD46 inactivates C3b and C4b in the initial steps of complement activation as a cofactor of CFI^50,51^. CD46 is constitutively expressed in glomerular capillaries^52^, suppression should lead to aggravation of complement-mediated injury.

miR-885-5p was one of the three downregulated miRNAs in glomeruli of DSA+. Targets include CASP3^53^, a key executor of apoptosis^54^. Interestingly, both miR-30d and miR-885-5p were increased in biopsies with glomerular C4d positivity compared to negative biopsies. Whereas miR-30d suppresses TP53^55^, miR-885 activates it^56^. The net effect of the upregulation of both miRNAs on TP53 upon complement activation is unclear, however, terminal complement activation has been reported to increase TP53 expression *in vitro* and *in vivo*^57^.

Our pilot study suggests that glomerulocapillary miRNAs could be valuable biomarkers for anti-HLA-class I DSA. Using the random forest method, we could identify 80-90% of biopsies correctly based on the glomerular miRNA expression, which clearly would not have been possible by conventional histopathology including C4d immunostainings. Random forest is a robust, automatic and cutting-edge prediction tool with minimum parameter tuning and does not overfit. It appropriately estimates the validation error rate during the analysis by the out-of-bag error rate, essentially carrying out training and validation by one step^58^. Random forest suited our low sample size, as it does not further reduce it by reserving a portion for validation. Nevertheless, before implementation as a routine diagnostic method prospective validation would still be required. Our method is complementary to conventional histology and can be used after routine histological workup, which would be advantageous compared to the already established methods which consume an entire biopsy core which is then lost for histopathology^59^. Yet, in order to improve ABMR-diagnostics our method will need refinement in prospective studies in order to identify glomerocapillary miRNAs indicative of impeding structural and functional deterioration with ABMR, not just reflecting the mere presence of HLA-class I DSA.

In summary, we have identified 12 glomerulocapillary miRNAs dysregulated with HLA-class I DSA. Their dysregulation seems to be detrimental, in particular proinflammatory, antiangiogenic and profibrotic. The precise function of these miRNA in endothelial cells should be further investigated, preferably in flow co-culture with other cellular mediators of ABMR. Moreover, our results from biopsy glomeruli are a first step towards the development of novel diagnostic tools for ABMR which could complement traditional histology. An analogous approach could be used to investigate the clinically more important effect of HLA-class II DSA, the combined effects of both HLA-class I and II DSA and of non-HLA-DSA on endothelial miRNA in more elaborate experiments which would require stable transfection of endothelial cells with HLA-class II or stimulation with IFNG. Finally, these glomerulocapillary miRNAs could be novel therapeutic targets in ABMR.

## Methods

### *In vitro* model of anti-HLA-class I alloantibody binding

Primary human renal glomerular endothelial cells (GECs) were cultivated according to the vendor’s instructions (4000; ScienCell, Carlsbad, CA, USA) in EBM medium (Lonza, Basel, Switzerland). Cells were used until passage 8. Proper endothelial differentiation was confirmed with quantitative real-time polymerase chain reaction (qRT-PCR) for endothelial markers KLF2, KLF4, VWF, ADAMTS13, CD31, CD46, CD55, CD59, THBD (data not shown); HLA-class I typing revealed HLA-A2 as target antigen. Pooled human anti-HLA-A2 serum served as anti-HLA-class-I, whereas irrelevant anti-HLA-A1 serum served as controls. Cells were serum starved with 0.2% fetal calf serum overnight. On the next morning, cells were incubated for 60 min in anti-HLA-class I or control serum, each diluted 1:1 with starvation medium. Cells were washed and then incubated for 60 min with rabbit complement (CABC-1D, One Lambda, Canoga Park, CA, USA). Thus, we obtained four cohorts: specific anti-A2 without complement as a model for anti-HLA-class I ABMR without complement activation (*in vitro* C−), specific anti-A2 with complement as a model for anti-HLA-class I ABMR with complement activation (*in vitro* C+) and the respective controls irrelevant anti-A1 without complement (*in vitro* control without complement) and irrelevant anti-A1 with complement (*in vitro* control with complement). Cells were harvested in trizol and RNA was isolated with the phenol-chlorophorm method. For each sample 350 ng RNA was reverse-transcribed with the Multi-Scribe-based High Capacity Kit and preamplified with the Megaplex RT stem-loop primer pool A and B, (Version 2.0, Life Technologies, Foster City, CA, USA). qRT-PCR was performed with Universal PCR Mastermix (Life Technologies) according to the user manual (A and B; v2.0, Life Technologies) enabling simultaneous quantification of 761 miRNA species. All qRT-PCR curves and respective C_T_ values were generated with RQ manager 1.2 (Life Technologies). Thresholds were set as 0.25. Each group was analysed in a separate RQ manager file. Detectable transcripts were defined as those with a C_T_ value below 40 and a regular sigmoid-shaped amplification curve. Amplification curves were subject to a stringent visual quality control. Baseline values were adjusted if necessary. All experiments were done in triplicates. 229 miRNAs detectable in less than 2 samples in all 4 groups were omitted from further analysis. 60 miRNAs, which were not detectable in at least one sample in all four groups were also omitted from further analysis. RNU48 and U6 snRNA served as reference transcripts. Relative glomerular expression was calculated as 2^(mean(C_T_^reference transcripts^)-C_T_^target^). The remaining 382 miRNAs (after omission of irrelevant species on the card) were screened for differential expression between *in vitro* C− or *in vitro* C+ vs. the respective control to discover complement-independent changes in miRNA expression upon anti-HLA-class I antibody binding and between *in vitro* C+ with complement vs. the respective control to discover changes in miRNA expression anti-HLA-class I antibody binding with complement activation.

### Selection of miRNAs for the confirmation in human transplant biopsies

We selected 15 miRNAs for validation in microdissected glomeruli of human transplant biopsies based on visual inspection of the volcano plots (Figs 1 and 2) and on *in silico* data about validated targets and pathways with miRPath^60^. To these 15 miRNAs we added miR-520e, although it was detected only in one sample of *in vitro* C+, because the predicted targets included complement regulator CD46.

### Confirmation in glomeruli of human transplant biopsies

Based on HLA-class I and II DSA data we selected a total of 30 indication biopsies from our archive; all were from different patients. The tests for anti-HLA-class I and II antibodies were performed with the LuminexTM technology (Luminex, Austin, TX, USA) using the LABScreen^TM^ Mixed Assay, One Lambda/Thermo Fisher, Canoga Park, CA) according to the manufacturer’s instructions. The discrimination of anti-HLA specificities in LABScreenTM mixed-positive samples was achieved with LABScreen^TM^ Single Antigen Beads (One Lambda/Thermo Fisher).All biopsies from patients with anti-HLA-class II DSA were excluded from the study. All 20 remaining biopsies with only HLA-class I DSA (DSA+) were divided into complement-binding and non-complement binding class I DSA with the C1q Screen assay (One Lambda). Four had only complement-fixating anti-HLA-class I DSA (C+), 6 had only complement-independent anti-HLA-class I DSAs (C−) and 10 had both complement-fixating and complement-independent anti-HLA-class I DSAs (C+/C−). Complement-fixating anti-HLA-class II DSAs not detectable in the standard Luminex assay were excluded in these patient by a negative lymphocyte cytotoxicity (LCT) tests.

Ten indication biopsies served as controls and were negative for anti-HLA-class I and II DSA based on negative standard Luminex tests and on negative LCT tests.

Clinical data were retrieved from the patients’ files. The estimated glomerular filtration rate (eGFR) was calculated according to the modification of diet in renal disease (MDRD) formula^61^ for all but one pediatric control patient, for whom we had to use the Schwartz formula^62^. Histological findings including the Banff components according to the most recent update^63^ were determined on our routine set of stainings including C4d on paraffin embedded tissue after epitope retrieval with citrate buffer at pH 6.0; glomerular C4d was reported in analogy to the Banff component C4d.

Glomeruli from the remaining tissue in the paraffin blocks were microdissected, glomerular miRNA was isolated and transcribed as recently described^64^ and quantified in analogy to the method described above.

### Statistical methods

For the comparison of our *in vitro* model for complement dependent and complement-independent effects of anti-HLA-class I DSA we drew volcano plots^65^. These plots show the log2 of the fold change in the mean relative expression on the x-axis and the -log10 of the p-value of a t-test comparing the replicates of the experiments on the y-axis.

All values are given as the median and the interquartile range. Continuous parameters were compared with Wilcoxon tests between two cohorts. P-values below 0.05 were considered significant in two-sided tests. However, in the retrospective analysis of biopsies, p-values can only be regarded as descriptive. In this explorative study we did not correct for multiple testing.

In order to assess the value of glomerular miRNA quantification for the discrimination between all patients with HLA-class I DSA and controls, a random forest analysis^66^ was performed. Random forest is an ensemble machine learning technique which generates many decision trees (e.g. 500) using a randomly chosen subset of predictive variables (e.g. 1/3 of them), on bootstrap (or sample with replacement) versions of the original data. The individual samples not used in the bootstrap version are nevertheless used in the calculation of the so-called “out-of-bag” (OOB) error estimation. The OOB error estimated is very similar to the leave-one-out cross-validation error rate, but these are obtained without an extra cost (without a collection of extra samples, as well as without extra computational work). Random forest is an attractive method for several reasons: it is a multivariate classifier and the contributions from these variables are non-additive; this method is much less prone to overfitting than others (e.g. single decision tree, regression, boosting, etc.); it evaluates the importance of each variable concerning its contribution to the classification performance. For more information on the pros and cons of random forest and comparison with other similar classifiers/predictors, see the review by Efron and Hastie^58^. The R (www.r-project.org) package randomForest was used with the default setting.

### Ethical approval

All transplants were performed according to the Declaration of Istanbul^67^. The study was performed in accordance with the Declaration of Helsinki^68^. Ethical approval was obtained from the Ethics Committees of Hannover Medical School and the University Hospital Essen with informed consent of the patients.

## Electronic supplementary material

Supplementary Dataset 1
