# Author Correction: Glomerulocapillary miRNA response to HLA-class I antibody *in vitro* and *in vivo*

**DOI:** 10.1038/s41598-018-23760-1

**Published:** 2018-04-12

**Authors:** Falko M. Heinemann, Peter T. Jindra, Clemens L. Bockmeyer, Philip Zeuschner, Juliane Wittig, Heike Höflich, Marc Eßer, Mahmoud Abbas, Georg Dieplinger, Katharina Stolle, Udo Vester, Peter F. Hoyer, Stephan Immenschuh, Andreas Heinold, Peter A. Horn, Wentian Li, Ute Eisenberger, Jan U. Becker

**Affiliations:** 1 Institute for Transfusion Medicine, University Hospital Essen, University Duisburg-Essen, Essen, Germany; 2 Immune Evaluation Laboratory, Department of Surgery, Baylor College of Medicine, Houston, TX USA; 3 Institute of Pathology, Department of Nephropathology, University Hospital Erlangen-Nürnberg, Erlangen, Germany; 4 Institute of Pathology, University Hospital of Cologne, Cologne, Germany; 5 Institute of Pathology, Hannover Medical School, Hannover, Germany; 6 PATHOCOM, Rheine, Germany; 7 Department of General, Visceral and Cancer Surgery, Transplant Center Cologne, University of Cologne, Cologne, Germany; 8 Children’s Hospital, Pediatrics II, University of Duisburg-Essen, Essen, Germany; 9 Institute of Transfusion Medicine, Hannover Medical School, Hannover, Germany; 10 Robert S Boas Center for Genomics and Human Genetics, Feinstein Institute for Medical Research, Northwell Health, Manhasset, NY USA; 11 Clinic for Nephrology, University Hospital Essen, University Duisburg-Essen, Essen, Germany

Correction to: *Scientific Reports* 10.1038/s41598-017-14674-5, published online 06 November 2017

The original version of this Article contained an error in the Abstract.

“Changes in miRNA expression glomerular of capillaries during antibody-mediated rejection (ABMR) are poorly understood and could contribute to the deleterious inflammation and fibrosis of ABMR via suppression of target genes.”

now reads:

“Changes in miRNA expression of glomerular capillaries during antibody-mediated rejection (ABMR) are poorly understood and could contribute to the deleterious inflammation and fibrosis of ABMR via suppression of target genes.”

This error has now been corrected in the PDF and HTML versions of the Article.

